# Ecosystem Health Evaluation and Ecological Security Patterns Construction Based on VORSD and Circuit Theory: A Case Study in the Three Gorges Reservoir Region in Chongqing, China

**DOI:** 10.3390/ijerph20010320

**Published:** 2022-12-25

**Authors:** Zhenzhen Yuan, Weijie Li, Yong Wang, Dayun Zhu, Qiuhong Wang, Yan Liu, Lingyan Zhou

**Affiliations:** 1 School of Geographical Sciences, Southwest University, Chongqing 400715, China; 2 School of Geographical Sciences, China West Normal University, Nanchong 637009, China; 3 School of Karst Science, Guizhou Normal University, Guiyang 550001, China

**Keywords:** ecological security pattern, ecosystem health, ecosystem services supply and demand, circuit theory, ecological corridor

## Abstract

Constructing ecological security patterns (ESPs) is an important approach to maintaining regional ecological security and achieving sustainable development. Most previous studies on ESPs mainly focused on the supply of ecosystem services (ESs) yet did not fully consider the ecosystem health and human demand for ESs, which lacked evaluation from the perspective of human nature. Therefore, based on ecosystem health and ESs demand, this paper constructed the “vigor, organization, resilience, ESs supply-demand ratio” (VORSD) ecosystem health evaluation system and combined it with circuit theory to develop a new and comprehensive ESPs identification framework. Taking the Three Gorges Reservoir Area in Chongqing section (TGRAC) as a case study, the results showed that the general ecosystem health of the TGRAC was not optimistic, and there was still a long way to go for ecological treatment and restoration. From the perspective of spatial distribution, there were significant differences in the ecosystem health between regions, and the eastern region was higher than the western region. The ecological sources area of the TGRAC was about 25,350.16 km^2^, mainly distributed in the northeast and southeast of forestland, grassland, and cultivated land. The total length of ecological corridors was 2291.41 km, linking the northeastern, southeastern, middle, and southwestern regions of the TGRAC. There were 82 ecological nodes and 30 ecological barriers, most of which were concentrated on the construction land and cultivated land in the southwest and should be regarded as priority areas for ecological conservation. The research results verify the regional suitability and rationality of integrating the VORSD model and circuit theory to construct ESPs, which can provide an important reference for regional ecological protection and land use pattern optimization.

## 1. Introduction

In recent years, with rapid socio-economic development and increased human activities, urban sprawl has greatly promoted the continuous transformation of the natural landscape into an artificial landscape, which has damaged the structure and function of ecosystems [1]. This intensification has caused a series of ecological problems, such as a decrease in soil erosion control, biodiversity and carbon storage, resulting in environmental degradation that affects regional sustainable development and ecological security [2,3,4]. As people pay more and more attention to the ecological environment and the sustainable development of human society, ecological security, the basis for ecosystems to provide various ESs for humans and guarantee ecosystem health, has been well developed in terms of its related theories and research methods [5,6,7], including ecological carrying capacity [8], ecological restoration [9], ecological security patterns [5,7] and nature reserve planning [10]. As an effective way to represent the health and integrity of ecosystems and alleviate the contradiction between ecological protection and sustainable socio-economic development, ecological security patterns (ESPs) have received extensive attention [11]. In addition, ESPs policy has become one of the important national strategies for ecosystem protection in China and is considered a bottom-line method for protecting key ecological areas [12]. Therefore, for developing countries, the construction of reasonable ESPs is the key issue of ecological civilization construction.

Based on numerous studies, ESPs consist of ecological sources, resistance surfaces, ecological corridors, and other key ecological elements [13,14,15]. Ecological sources are patches that are important for biodiversity maintenance [16], which are the basis for constructing ESPs. Identifying ecological sources has usually been achieved by directly selecting natural reserves [17] and land use types with high ecological value [18] or establishing a comprehensive ecological index evaluation system based on ESs [19], ecosystem sensitivity [20], and landscape connectivity [21]. According to the eco-environmental status and ecological problems of the study area, ESs were selected from the supply, regulation, support, and cultural services for superposition analysis, which has been the most commonly considered method for identifying ecological sources [22]. The ultimate purpose of ESPs is to ensure that ecosystems provide services in a sustainable and healthy state to meet human demands [23]. However, most studies only considered the supply of ESs but ignored the impact of ecosystem health and ESs demand on ecological sources identification [24,25], which was difficult to guarantee regional ecological security and sustainable development to a certain extent. The “vigor, organization, resilience, ESs supply” (VORS) model has been widely used to evaluate ecosystem health by considering ecosystem structure, function, and integrity [26,27]. From the perspective of indicator content, the VORS model emphasizes the ability to continuously provide ESs without considering the ESs demand of humans [28]. Ecosystems have gradually evolved into the nature-society-economy compound ecosystem [29], and with humans as the ecosystem’s important constituent, the demand for ESs will directly or indirectly affect the quality of the ecological environment. In addition, the supply-demand relationship of ESs reflects the result of the interaction between ecosystems and human activities. Therefore, this study integrated ecosystem integrity and the ESs supply and demand into the ecosystem health evaluation framework [28] and identified ecological sources according to the evaluation results.

Ecological resistance surface construction is an important basis for identifying ecological corridors [16]. In an ecosystem, the transfer of matter and energy and the migration of species are affected by land use types, environmental factors, and human activities [30,31]. Previous studies have assigned different resistance values for different land use types, which easily ignored the internal differences within the same land use type [32]. Thus, researchers have attempted to introduce nighttime light data [20], impervious surface coefficient [33], and habitat quality index [5] to modify ecological resistance surfaces. Ecological corridors are important links to maintain the flow of matter, energy, and information within ecological sources and can effectively maintain the mobility and diversity of species [34]. The minimum cumulative resistance (MCR) model was most widely used to identify ecological corridors [20,35]. However, although the MCR model can indicate the optimal route, it cannot reflect the random movement of species and fails to directly clarify the range and nodes of ecological corridors [5]. The circuit theory simulates species migration and identifies key nodes according to current intensity based on the characteristics of the random pathway of electrons in a circuit [36], so it can compensate for the above-mentioned deficiencies of the MCR model to identify ecological corridors.

The TGRAC has the characteristics of a reservoir and mountainous area and is an ecological barrier in southwest China and one of the main ecologically sensitive zones in the upper reaches of the Yangtze River [20]. However, under the pressure of rapid economic development and urbanization, coupled with the fragility of the ecological background, the ecological security of the TGRAC is facing serious threats. This study took the TGRAC as a case study, and the following research objectives were proposed: (1) to evaluate regional ecosystem health and identify ecological sources by the VORSD model in the establishment of the human-nature coupled system; (2) to establish ecological resistance surfaces based on habitat quality index, and to extract ecological corridors based on the circuit theory, so as to construct regional ESPs; (3) to provide suggestions for the ecological protection of the Three Gorges Reservoir area according to the construction of ESPs. Overall, this study can provide a new research framework and reference for ecological protection management and territorial space optimization in ecologically fragile regions.

## 2. Study Area and Data Sources

### 2.1. Study Area

**Figure 1:**
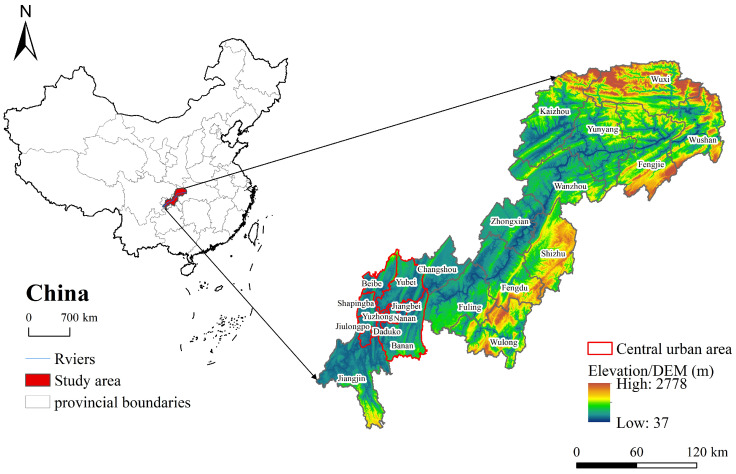
Geographical location and overview of the study area.

The TGRAC (28°31′–31°44′ N, 105°49′–110°12′ E) is located at the end of the Upper Reaches of the Yangtze River in southwest China, with a total area of about 46,163.67 km^2^ [20]. The TGRAC comprises 22 districts and counties, including the central urban area of Chongqing municipality (Figure 1). The topography of the TGRAC is complex, with a height difference of more than 2700 m, and the main geomorphological types are hills and mountains. The climate is subtropical temperate, with annual precipitation of about 1150 mm. In addition, there are 27 nature reserves that provide not only suitable habitats for wildlife but also have great potential for carbon sequestration [37]. The region contains ecological function zones and has a prominent and strategic ecological position in China, including regions of water and soil conservation in the TGRAC, high biodiversity in Qinling-Daba, and the Wuling Mountain areas. The ecological function of soil conservation was significantly enhanced, but the area of soil erosion was about 34% of the total area. By the end of 2020, the urbanization rate of the TGRAC was 64.68%, and the GDP accounted for 67.64% of the total output value. Land use types in the TGRAC were dominated by cultivated land, forestland, and grassland, which accounted for 42.69%, 38.78%, and 13.94% of the total area, respectively. There is no doubt that population growth and urban expansion have accelerated the transformation from natural to urban landscapes and exacerbated the fragility of the ecological environment.

### 2.2. Data Sources

The spatial and statistical datasets were used in this paper. Spatial datasets included the following: (1) Land-use data with a resolution of 30 m in 2015 were derived from the Resource and Environmental Science and Data Center of the Chinese Academy of Sciences (http://www.resdc.cn, accessed on 18 November 2021). (2) The DEM (30 m) was supported by the Geospatial Data Cloud Platform (https://www.gscloud.cn/, accessed on 16 November 2021). (3) The evapotranspiration data (1000 m) was obtained from the Global Drought and Potential Evapotranspiration Database (http://www.cgiar-csi.org/data/global-aridity-and-pet-databas, accessed on 27 December 2021). (4) The normalized difference vegetation index (NDVI) and population density were also obtained from the Resource and Environmental Science and Data Center of the Chinese Academy of Sciences (http://www.resdc.cn, accessed on 16 November 2021 and 13 November 2021, respectively). (5) The annual average air temperature and annual precipitation (1000 m) were derived from the National Earth System Science Data Center (http://www.geodata.cn/data, accessed on 12 May 2021). (6) Soil datasets came from the National Qinghai-Tibet Plateau Scientific Data Center (https://data.tpdc.ac.cn/zh-hans/, accessed on 27 December 2021). (7) The nature reserves data came from the China Nature Reserve Specimen Resources Sharing Platform (https://www.papc.cn/, accessed on 6 October 2021). (8) The statistical data on water consumption, grain consumption, and carbon emission came from the Water Resources Bulletin, the Chongqing Statistical Yearbook, and the China Emissions Inventories and Datasets (CEADs), respectively.

## 3. Research Methods

**Figure 2:**
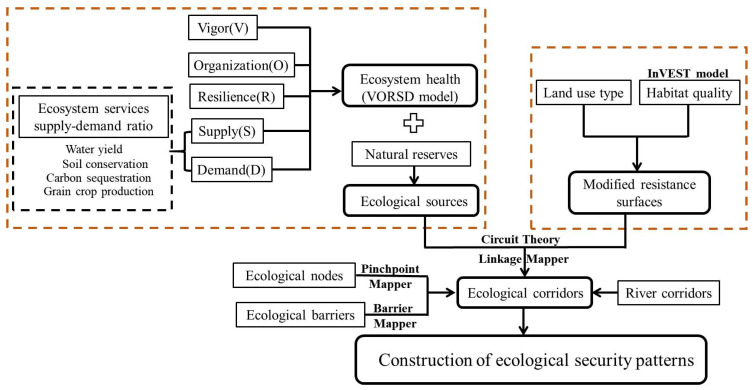
Research framework.

The focus of this study was to evaluate regional ecosystem health and construct ESPs, which mainly included the following steps (Figure 2). Firstly, the “vigor, organization, resilience, ESs supply-demand ratio” (VORSD) ecosystem health evaluation system was used to quantify the status and sustainability of ecosystem health. In this study, combined with the ecological environment and problems of the TGRAC, four evaluation indexes of water yield, soil conservation, carbon sequestration, and grain crop production were selected to evaluate the importance and demand of the ESs. Secondly, based on the results of ecosystem health evaluation, ecological patches with high ecosystem health levels and nature reserves were recognized as ecological sources. Thirdly, we quantified the habitat quality index based on the Integrated Valuation of Ecosystem Services and Trade-Offs Model (InVEST model) and used its inverse to modify resistance surfaces that were assigned for land use types. Finally, the circuit theory was established to identify ecological corridors, nodes, and barriers, thus constructing ESPs of the TGRAC. A detailed explanation of each step is described in the following sections.

### 3.1. Ecosystem Health Evaluation

The important purpose of ESPs research is to ensure that ecosystems can provide services in a sustainable and healthy state [38]. Therefore, based on the traditional VORS evaluation framework [27], this study fully considered ESs demand and used the ESs supply-demand ratio to replace ESs supply to jointly construct the “vigor, organization, resilience, ESs supply-demand ratio” (VORSD) ecosystem health evaluation system [28]. The specific calculation method of the ecosystem health index is as follows:

(1) $$EHI=\sqrt[{4}]{EV\times EO\times ER\times CESDR}$$

where *EHI* is the ecosystem health index; *EV*, *EO*, and *ER* represent ecosystem vigor, ecosystem organization, and ecosystem resilience, respectively; *CESDR* is the comprehensively supply-demand ratio of ESs. All indicators need to be standardized before the calculations to eliminate differences in dimensions [1]. The standardized formula is as follows:

(2) $$\mathrm{Positive}\;\mathrm{indicators}:\;y_{j}=\left(x_{j}-x_{j\min}\right)/\left(x_{j\max}-x_{j\min}\right)$$

(3) $$\mathrm{Negative}\;\mathrm{indicators}:\;y_{j}=\left(x_{j\max}-x_{j}\right)/\left(x_{j\max}-x_{j\min}\right)$$

where *y_j_* is the standardized value of *x_j_*; *x_j_* is the original value of indicator *j*; *x*_*j*max_ and *x*_*j*min_ represent the maximum value and minimum value of indicator *j*, respectively.

#### 3.1.1. Ecosystem Physical Health

Ecosystem physical health refers to the integrity of ecosystem components, structure, and function, which is often measured by three indicators: *EV*, *EO*, and *ER* [39]. *EV* is defined as the metabolic capacity or primary productivity of an ecosystem [40]. NDVI was widely selected to characterize the *EV* because of its positive correlation with vegetation productivity and its ability to monitor eco-environmental quality effectively [41]. *EO* refers to the stability of ecosystem structure, which mainly reflects in landscape heterogeneity, landscape connectivity, and the connectivity of patches with important ecological functions [37] (Table 1). The relative landscape index was calculated by Fragstats4.2, and combined with the research of Peng et al. [27], the weight of the landscape index was set. The Shannon Diversity Index (*SHDI*) and area-weighted mean patch fractal dimension (*AWMPFD*) were used to measure landscape heterogeneity. The overall landscape connectivity was indicated by the landscape fragmentation index (*FN*_1_) and landscape contagion index (*CONT*). Forestland, grassland, and water body are important media to connect other patches and landscapes. These land types had important ecological functions, providing a variety of ecological services that were indispensable for human survival and development. Three important ecological patches of forestland, grassland, and water were measured by the fragmentation index (*FN*_2_, *FN*_3_, *FN*_4_) and cohesion index (*CONHES*_1_, *CONHES*_2_, *CONHES*_3_). *ER* refers to the stability of ecosystem structure and function under external disturbance [27], including resistance and resilience to external disturbance (Table 1). Resilience and resistance coefficients with different weights and levels were allocated according to land use types [27,42].

#### 3.1.2. Ecosystem Services Supply and Demand

Ecosystem services are the benefits humans derive directly or indirectly from ecosystems [43]. Considering the actual situation of the ecosystem and the availability of data in the TGRAC, four typical ecosystem services were selected to reveal the quantitative relationship and spatiotemporal distribution pattern of the ESs supply and demand [28].

1. Water yield

The TGRAC is of great significance in keeping the balance of water supply and demand for regional ecosystem stability. According to Liu et al. [44], the Water Yield module of the InVEST model was used to calculate the water supply, and the demand was expressed in terms of actual water consumption, including industrial, agricultural, and domestic water, and calculated by the product of per capita water consumption and population density [44,45]. The calculation formulas are as follows:

(4) $$\mathrm{Supply}:\;S_{wp}=\left(1-\frac{AET_{x}}{P_{x}}\right)\times P_{x}$$

(5) $$\mathrm{Demand}:\;D_{wx}=D_{perwater}\times D_{xpop}$$

where *S_wp_* represents the annual average water yield of grid cells (m^3^/hm^2^); *P_x_* is the average annual precipitation at pixel *x* (mm); *AET_x_* is the actual annual evapotranspiration at pixel *x* (mm). *D_wx_* represents the water demand of grid *x* (m^3^/hm^2^); *D_perwater_* is the annual average water consumption, and *D_xpop_* is population density at pixel *x*.

2. Soil conservation

The TGRAC is mainly mountainous and hilly, with undulating terrain. In recent decades, the intensity of human exploitation has been increasing, which has affected surface vegetation coverage and resulted in the frequent occurrence of soil erosion. Soil conservation was used as the supply of soil conservation services, and the demand was measured by the actual amount of soil erosion that humans expected to cause [45]. The formulas are as follows:

(6) Supply: SEDRET=RKLS−USLE=R×K×LS×(1−C×P),

(7) Demand: USLE=R×K×LS×C×P,

where *SEDRET* is the soil conservation amount (t/hm^2^); *RKLS* represents potential soil erosion (t/hm^2^); *USLE* represents the actual soil erosion (t/hm^2^); *R* is the rainfall-runoff erosion factor; *K* is the soil erosion factor; *LS* is the slope steepness and length; *P* is the soil and water conservation factor, and *C* is the vegetation cover factor.

3. Carbon sequestration

The balance between supply and demand for carbon sequestration services is of great significance for mitigating climate change and protecting the regional atmospheric environment. According to Zhu et al. [46], the Carbon Storage module of the InVEST model was used to measure the supply. The carbon storage of the ecosystem (*C_tot_*) was divided into four basic carbon pools from different land use types [47], including the aboveground biological carbon (*C_above_*), belowground biological carbon (*C_below_*), soil organic carbon (*C_soil_*), and dead organic carbon (*C_dead_*). The demand was expressed as the carbon emissions generated by human energy consumption [45]. The calculation formulas are as follows:

(8) $$\mathrm{Supply}:\;C_{tot}=C_{above}+C_{below}+C_{soil}+C_{dead},$$

(9) $$\mathrm{Demand}:\;D_{cx}=D_{percarbon}\times D_{xpop},$$

where *C_tot_* represents the total carbon storage (t/tm^2^); *D_cx_* represents the total carbon emissions of grid *x* (t/tm^2^); *D_percabon_* is the annual average carbon emissions.

4. Grain crop production

Grain crop production is an important service provided by the agricultural ecosystem [24]. Previous studies have shown a significant linear relationship between crops and NDVI [48]. Therefore, the supply was evaluated according to the ratio of the grid NDVI value relative to the total cultivated land NDVI value [49]. The demand was multiplied by the population density to obtain the grain demand of each grid [50]. According to the per capita food demand standard of Chongqing published by the National Bureau of Statistics, the annual grain crop consumption per capita demand was 382.85 kg. The calculation formulas are as follows:

(10) $$\mathrm{Supply}:\;G_{x}=G_{sum}\times \frac{{NDVI}_{x}}{{NDVI}_{sum}},$$

(11) $$\mathrm{Demand}:\;D_{cpx}=D_{percrop}\times D_{xpop},$$

where *G_x_* represents the annual average grain crop production at pixel *x* (t/tm^2^); *G_sum_* is the total grain yield; *NDVI_x_* is the normalized vegetation index of grid *x*; *NDVI_sum_* is the sum of NDVI values; *D_cpx_* represents the demand of grain crops of grid *x* (t/tm^2^); *D_percrop_* is the annual average grain crop consumption.

5. Ecosystem services supply-demand ratio

The ecosystem services supply-demand ratio (*ESDR*) can reflect the quantitative and spatial relationships of ESs supply and demand as well as the ability of ecosystems to sustainably provide ESs [51]. A positive value of *ESDR* means a surplus, zero means balance, and a negative value indicates a deficit state [24].

(12) $$ESDR=\frac{S-D}{\left(S_{\max}+D_{\max}\right)/2},$$

where *S* and *D* refer to the supply and human demand of ESs; *S*_max_ and *D*_max_ are the maximum supply and human demand of ESs, respectively.

6. Comprehensively ecosystem services supply-demand ratio

Comprehensively ecosystem services supply-demand ratio (*CESDR*) reflects the overall supply-demand relationship and spatial distribution of ESs in the study area [44]. The calculation formula is as follows:

(13) $$CESDR=\frac{1}{n}\sum_{i=1}^{n}ESDR_{i},$$

where *ESDR_i_* is the supply-demand ratio for type *i* ESs; *n* is the number of ecosystem services types, *n* = 4 in this paper.

### 3.2. Identification of Ecological Sources

Ecological sources are the ecological areas that can provide diverse ESs and have high levels of ecosystem health [37]. Previous studies rarely paid attention to the impact of ecosystem health and ESs demand on ecological sources identification [24,25], ignoring that the fundamental purpose of ESPs research is to ensure that ecosystems can provide services in a sustainable and healthy state [38]. Therefore, according to the evaluation results of the ecosystem health status of the TGRAC, the ecological source areas were identified. Based on the natural breakpoint method, the results of ecosystem health evaluation were divided into five grades [25]: well, relatively well, ordinary, relatively weak, and weak. The well and relatively well ecological patches were regarded as the candidate areas of ecological sources. Consider that ecological sources must have a certain area to make the core area isolate from external interference [37], and refer to previous research results of the same scale [20], the spatially continuous patches with areas of larger than 10 km^2^ were selected as ecological sources. Nature reserves provide places for species to survive and migrate, so these areas should be included in the scope of ecological sources [25].

### 3.3. Construction of Resistance Surface

The resistance surface reflects the hindrance degree of landscape heterogeneity on species migration [7] and is greatly influenced by land use types and human activity intensity. Therefore, according to the comprehensive consideration of the TGRAC land-use status and existing studies [32], this study determined the resistance values of different landscape types and constituted the basic ecological resistance surface. Habitat quality represents the suitability of the regional environment and biodiversity [7]. Habitat quality was calculated by the InVEST model, and its inverse was used to modify the basic ecological resistance surface to describe the differential effects in the same land use type on species migration. The influence of social factors, such as human activity intensity, has been taken into account in the calculation of habitat quality, which can reduce the interference of subjective factors and obtain more scientific ecological resistance surfaces.

(14) $$R=\frac{1}{Q_{xj}},$$

(15) $$Q_{xj}=H_{j}\left[1-\left(\frac{D_{xj}^{z}}{D_{xj}^{z}+K^{z}}\right)\right],$$

where *R* represents the ecological resistance value; *Q_xj_* is the habitat quality of grid *x* in land use type *j*; *H_j_* is the habitat suitability of land use type *j*; *D_xj_* is habitat degradation degree of grid *x*; *K* is the half-saturation constant and *z* is the default parameter size.

### 3.4. Extraction of Ecological Corridors, Nodes, and Barriers

The construction of ecological corridors can improve the connectivity between patches or landscapes to optimize ecosystem functioning, reduce landscape fragmentation and promote regional ecological security [34]. Based on circuit theory, ecological sources were regarded as the focal nodes of the circuit, and ecological resistance surfaces were considered the conductive surfaces [7]. In this study, the Linkage Mapper was used to extract ecological corridors. The ecological processes of species in the landscape were simulated by the electronic random walk [34]. The least cost path (LCP) can be identified as the optimal path of species migration, which reflects ecological corridors. Ecological nodes were areas with high current density within ecological corridors. These nodes had important landscape connectivity and were key locations for preventing habitat degradation [52]. The Pinchpoint Mapper tool was used to identify the cumulative current values in the TGRAC, where the top 15% of cumulative current values were selected as ecological nodes. Ecological barriers were areas where resistance would reduce the landscape connectivity between ecological sources and affect species migration. The Barrier Mapper tool was used to identify the improve score and spatial distribution of obstacle points. The identified improve score was converted into low-level, medium-level, and high-level restoration areas according to the natural breakpoint method [53], where the high-level restoration areas were classified as ecological barriers.

## 4. Results

### 4.1. Ecosystem Health Evaluation

#### 4.1.1. Evaluation of Ecosystem Physical Health

**Figure 3:**
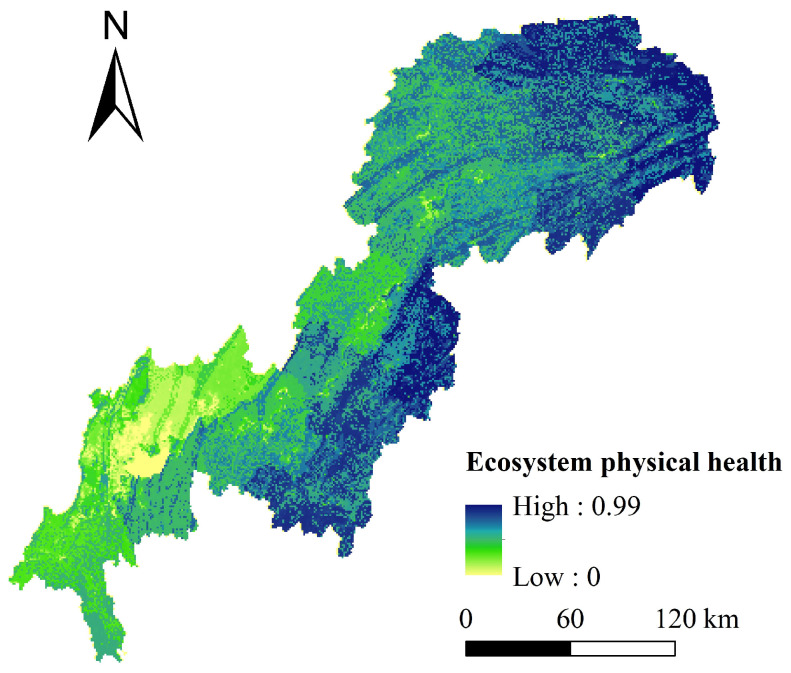
Spatial distribution of ecosystem physical health.

The average value of the ecosystem physical health index was 0.67, and most areas were dominated by medium and low values. High-value areas were mainly distributed in forestland, grassland, and cultivated land in the northeast and southeast of the TGRAC (Figure 3). Low-value areas were concentrated in parts of the southwestern and central of the study area. Because of the relatively high-intensity human activities, natural landscapes were gradually taken over by artificial landscapes, which reduced landscape connectivity and weakened the resistance and resilience of ecosystems.

#### 4.1.2. Evaluation of Ecosystem Services Supply and Demand

**Figure 4:**
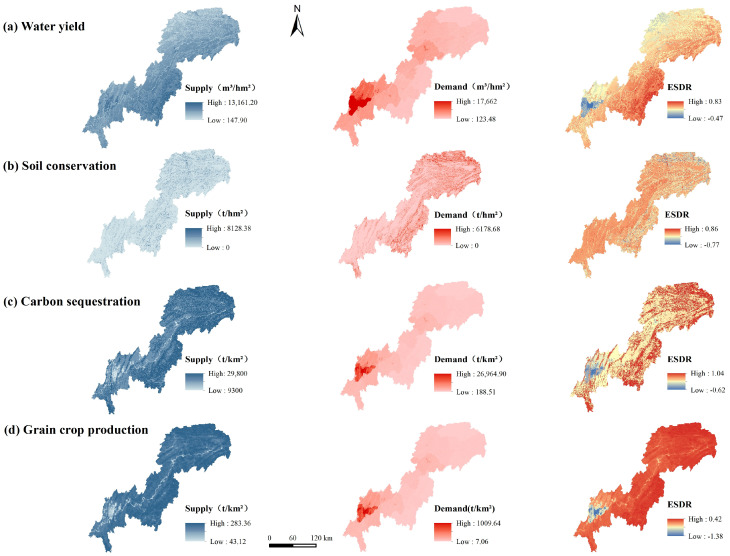
Spatial distribution of supply and demand of ESs.

The total supply of water yield service was 3.28 × 10^8^ m^3^, higher than the total water demand of 4.64 × 10^7^ m^3^. High-supply areas were mainly distributed in the southwest and southeast of the TGRAC (Figure 4a). The high level of urbanization in the southwest has increased impervious surfaces, which reduced surface infiltration and soil moisture. High supply was located in southeastern TGRAC due to more precipitation than in other regions. High-demand areas were mainly located in central urban areas and other district or county centers. Overall, water yield in most regions was in surplus, with the deficit mainly located in central urban areas owing to high population density.

The average supply of soil conservation service was 80.68 t/hm², and the average demand was 176.09 t/hm². The high supply areas were mainly distributed in mountainous areas with high vegetation coverage, which showed a “northeast-southwest” stripe distribution (Figure 4b). The high-demand areas were concentrated in Wushan, Wuxi, and Fengjie. In general, more than 60% of soil conservation was in surplus.

The total supply of carbon sequestration service was 9.6 × 10^8^ t. The supply showed a decreasing spatial distribution trend from east to west in the TGRAC (Figure 4c). High-supply areas were mainly distributed in the Daba Mountain, Wushan Mountain, and Wuling Mountain areas, which were important carbon sinks and oxygen sources with high vegetation coverage. However, due to high population density and large carbon emissions from industry and automobiles, the demand was high, and the ESDR was in deficit in the central urban area of Chongqing.

The total supply of grain crop production was 1.15 × 10^7^ t, with an average supply of 251.33 t/km². High supply was concentrated in the southeastern and northeastern regions (Figure 4d), which had relatively large cultivated land resources. The spatial distribution of the demand was similar to population density, with high values mainly in densely populated districts such as Yuzhong, Shapingba, Jiulongpo, and Jiangbei. The deficit was extremely high in the central urban area of Chongqing municipality due to lower cultivated lands and large populations.

#### 4.1.3. Spatial Pattern of Ecosystem Health Evaluation

**Figure 5:**
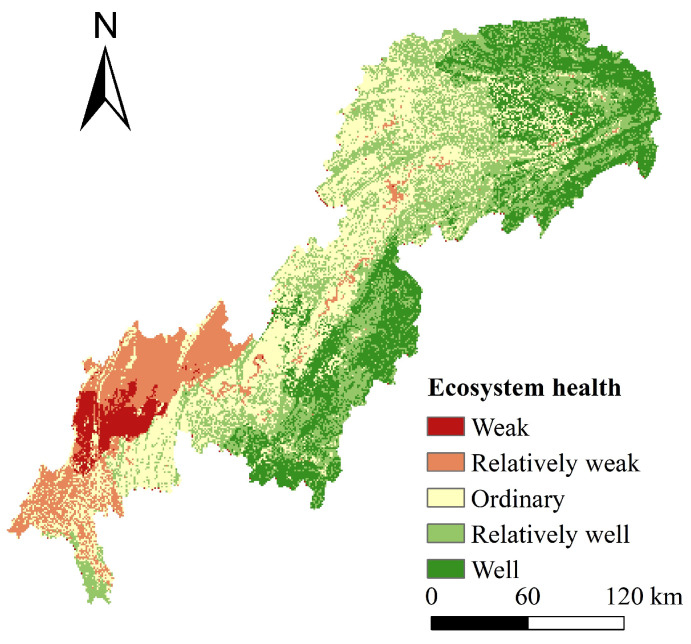
Spatial distribution of ecosystem health evaluation.

In general, the spatial distribution of ecosystem health was high in the east and low in the west (Figure 5). Central urban areas and other district or county centers of the TGRAC had low ecosystem health values, caused by the gradual transformation from natural ecosystems to semi-natural and artificial ecosystems, with high construction land density and low vegetation coverage. In the northeast and southeast of the TGRAC and the south of Jiangjin District, the overall ecological background was categorized as well because of high vegetation coverage and the low population density, which had the ability of sustainable high supply to meet the ecological demand of humans. According to statistics, the well-priority area covered 9529 km^2^, accounting for 20.64% of the total area of the TGRAC. The weak-priority and relatively weak-priority areas covered 14,855 km^2^ or 32.18% of the TGRAC, indicating that the overall ecosystem health of TGRAC was not optimistic and ecological environment construction was still facing great pressure, especially in the southwest of the study area.

### 4.2. Ecological Sources Identification

**Figure 6:**
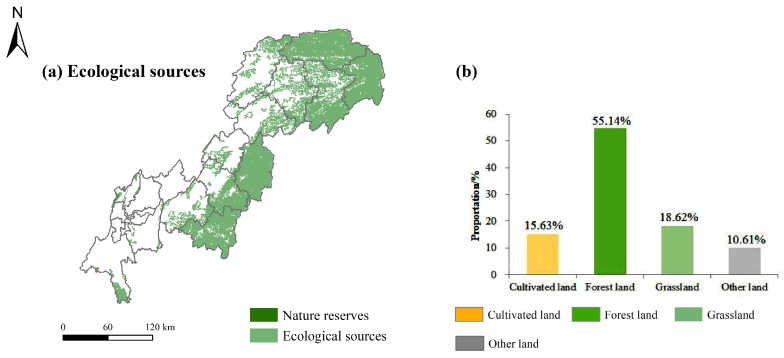
Spatial distribution of ecological sources (**a**), and composition of land use types in the ecological sources (**b**).

A total of 95 ecological sources were identified, with a total area of 25,350.16 km^2^, accounting for 54.91% of the TGRAC. The ecological sources were mainly distributed in the south of Daba Mountain, the west of Wushan Mountain, Wuling Mountain in the southeast of the TGRAC, Simian Mountain, and Jinyun Mountain (Figure 6a), with dense vegetation coverage and less impact from human activities. Almost no ecological sources were detected in Jiangbei, Yuzhong, Na’nan, Jiulongpo, Daduhe, and Shapingba within the central urban areas because of the landscape fragmentation. From the viewpoint of land use types, ecological sources consisted of forestland, grassland, and cultivated land, which accounted for 55.14%, 18.62%, and 15.63% of the total area of ecological sources, respectively (Figure 6b). In terms of the spatial distribution of administrative regions, Wuxi had the largest proportion of ecological source areas, followed by Wushan, which accounted for 15.81% and 14.38% of the total area of ecological sources, respectively.

### 4.3. Resistance Surfaces Construction

**Figure 7:**
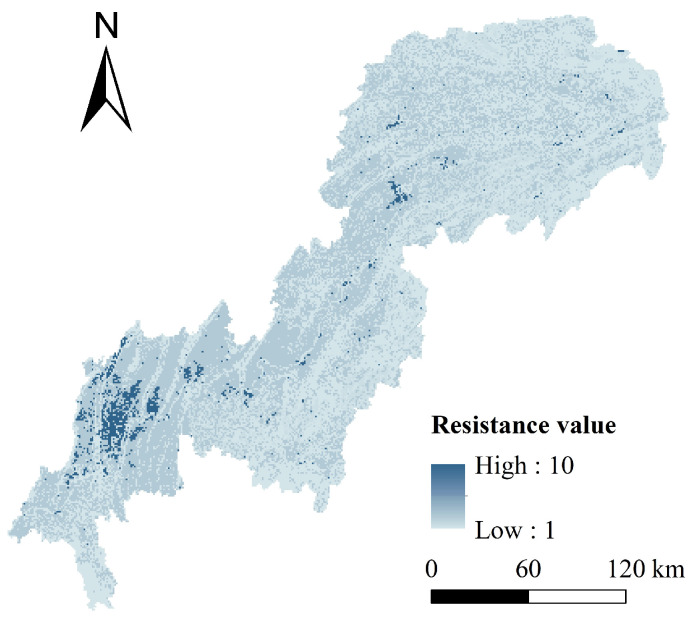
Spatial distribution of resistance surfaces.

The average ecological resistance value of the TGRAC was 1.59. The areas with high resistance were primarily distributed in the central urban areas and the built-up areas of other counties or districts, while the resistance values in most other areas were low (Figure 7). Yuzhong and Jiangbei had the highest ecological resistance value, with a value of 8.47. Wuxi and Wushan had the lowest ecological resistance value at 1.28. Human activities were relatively frequent in urban areas, where the disturbance of ecosystems was obvious. This disruption has resulted in intensified habitat fragmentation and increased resistance to the connectivity of ecological patches.

### 4.4. Ecological Nodes and Corridors Extraction

**Figure 8:**
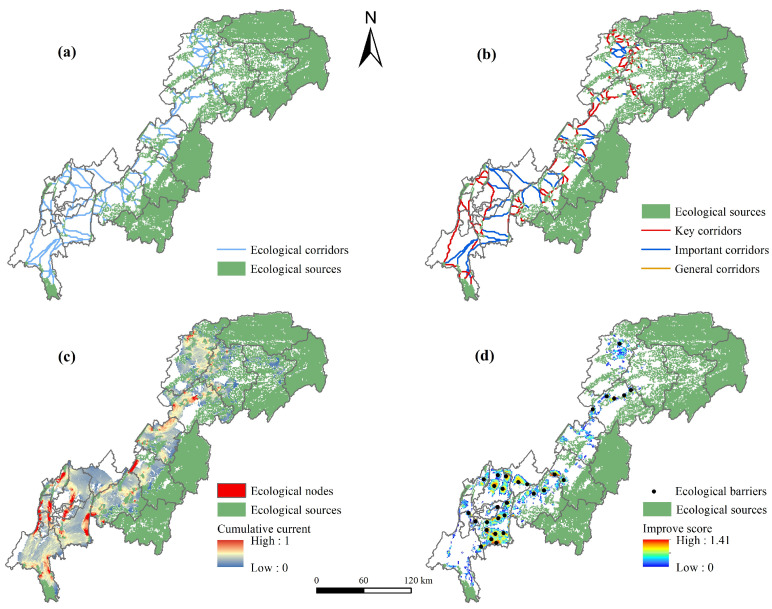
Spatial distribution of ecological corridors (**a**), corridors classification (**b**), nodes (**c**), and barriers (**d**).

A total of 201 ecological corridors were extracted, with an average length of 11.40 km. From the perspective of the degree of interconnection between ecological sources, most of the corridors were uniformly distributed in the western and central parts of the TGRAC (Figure 8a), which can effectively enhance the ecosystem connectivity and stability of ecological sources. Note that ecological sources were more dispersed and required longer corridors to maintain their mutual connectivity in the southwest. In addition, calculating the ratio of the cost-weighted distance of ecological corridors to the length of the path of corridors can reflect the relative resistance of species to migration. The relative resistance of corridors ranged from 6.08 to 208.26, with an average of 25.28. Corridors were divided into key corridors, important corridors, and general corridors by the natural breakpoint method [25] (Figure 8b). The number of key corridors was 153, accounting for 76.12% of the total corridors, mainly distributed in Kaizhou, Wanzhou, Zhongxian, Fengjie, and Jiangjin. There were 57 important corridors, most of which were distributed among the ecological sources far apart in the southwest.

Ecological nodes were modeled in the “all-to-one” model based on circuit theory, and the high cumulative current values (top 15%) in the red area were considered ecological nodes (Figure 8c). There were 82 ecological nodes with a total area of 629 km^2^. It can be found that all of the nodes were distributed on the key ecological corridors, which become obstacles affecting landscape connectivity. In terms of land use types, ecological nodes were mostly distributed in construction land and cultivated land in the southwest of the TGRAC. These areas were heavily exploited by human activities, resulting in high potential ecological risks to the surrounding areas [54].

The improve score was between 0 and 1.41 (Figure 8d), with values representing the importance of ecological restoration. The red areas represented barriers to landscape connectivity and were urgently in need of improvement [53]. In total, 30 ecological barriers were identified, mainly in Banan, Northern Yubei, Wanzhou, and the border between Fuling and Fengdu. These regions were important economic towns with relatively intense human activities, resulting in fewer and scattered ecological sources [20]. The improve score of barriers was divided into low-level, middle-level, and high-level restoration areas of 2216 km², 1491 km², and 826 km², respectively. The low-level restoration areas were mainly concentrated in forestland and cultivated land. The high-level restoration areas were mainly distributed in cultivated land, water areas, and construction land. Obviously, although the high-level restoration areas had great repair value, it was difficult and costly to repair.

## 5. Discussion

### 5.1. Comparison of VORSD and VORS Models

The identification of ecological sources is one of the key difficulties in the construction of ESPs, and scholars have made a lot of studies and attempts at source identification [5,7,20]. Previous studies largely emphasized the single measurement of an ecosystem’s natural state (ESs, ecosystem sensitivity, or landscape connectivity) to identify ecological sources. However, there is still a lack of adequate consideration of ecosystem health and ecological demand, which is not conducive to the identification of ecological sources from the perspective of coupled human and ecological systems [28]. Therefore, this paper identified ecological sources by integrating ecosystem health, ESs supply, and ecological demand and emphasized ecological background and the interference effects of human factors on ecosystems. Firstly, ecosystem health can directly and comprehensively reflect the quality of regional ecosystems [26], and ecological patches with a high level of ecosystem health are supposed to be ecological sources [37]. Secondly, ecological supply reflects the ability of natural systems to continuously provide ESs to humans, and ecological source patches should have a higher supply of ESs than other patches [7]. Thirdly, as an important part of an ecosystem, ecological demand is also the embodiment of the impact of human activities. If the region has the natural background conditions to provide high ecosystem health and ESs, it cannot meet the ecological demand of humans, and it is impossible to maintain biological diversity and the stability of ecological processes in the long term [24]. Based on the traditional VORS evaluation framework, the ecological demand was fully considered, and the VORSD ecosystem health evaluation framework was constructed to identify ecological sources.

**Figure 9:**
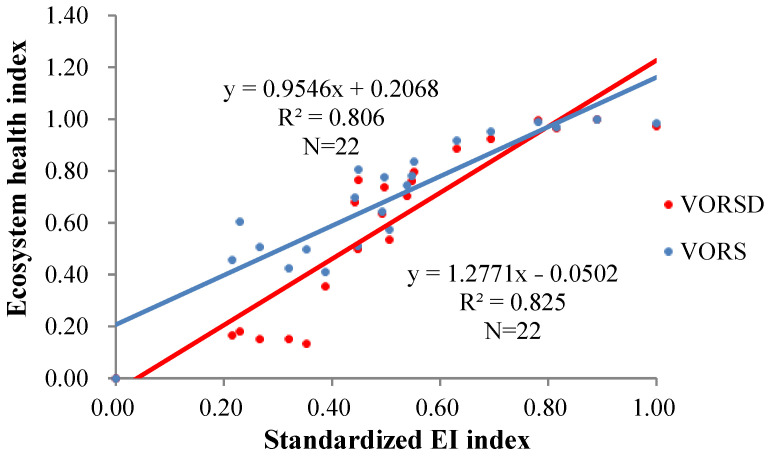
The regional adaptation assessment results for VORS and VORSD models using the standardized EI index.

In order to verify the regional adaptability and simulation accuracy of VORSD and VORS models, the evaluation results of each model were compared and analyzed with the National county Ecological Index (Figure 9). The ecological index (EI) was used to evaluate the overall status of the regional ecological environment quality, which was subject to the official announcement of the Chongqing Ecological Environment Bureau. In terms of model accuracy, it can be found that the VORSD model (0.825) was significantly higher than the VORS model (0.806). At the regional scale, the VORSD model can better reflect the actual situation of the ecosystem and was significantly better than the VORS model.

**Figure 10:**
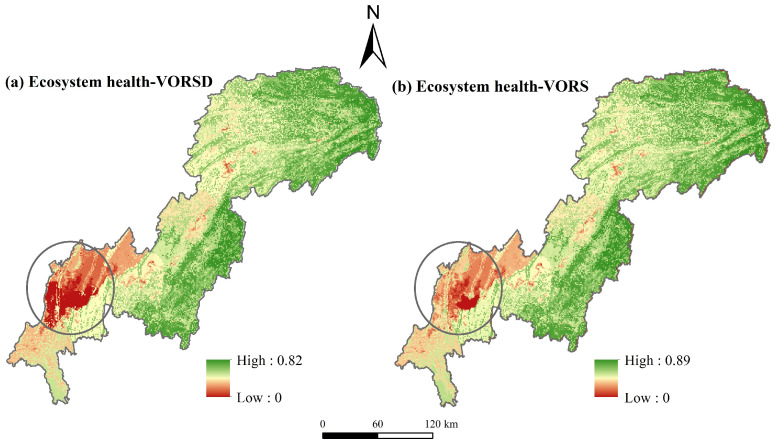
Spatial distribution of ecosystem health based on the VORSD model (**a**) and VORS model (**b**).

The differences in ecosystem health calculated by the two models are reflected in Table 2. The results showed that the standard deviation, average, median, and maximum values of the VORSD model were slightly lower than those of the VORS model. The results of the VORS model were polarized and difficult to reflect the internal ecosystem health differences, while the VORSD model showed a state of uniform distribution. This is because the VORSD model used the ratio of supply-demand of ESs to replace the supply of ESs [28], which made the ecosystem health level in some regions with the deficit of ESs supply and demand lower than the evaluation result of the VORS model, especially in areas with rapid urbanization and their surrounding areas (Figure 10). The spatial distribution in low ecosystem health-level regions was wider (Figure 10). The results were consistent with Pan et al. [28]. As the core of the economic and urbanization development of the TGRAC, the southwest central urban areas were severely affected by human activities, and the land use type was still mainly construction land, which was difficult to provide sustainable ESs and high levels of ecosystem health. The VORSD model can not only better reflect the actual situation of the ecosystem but also take the welfare of humans into consideration and has intense sensitivity in identifying regions with high-intensity human activities or a high degree of landscape fragmentation. Thus, it is more reasonable to identify ecological sources based on the VORSD model.

### 5.2. Ecological Security Patterns Construction and Protection

**Figure 11:**
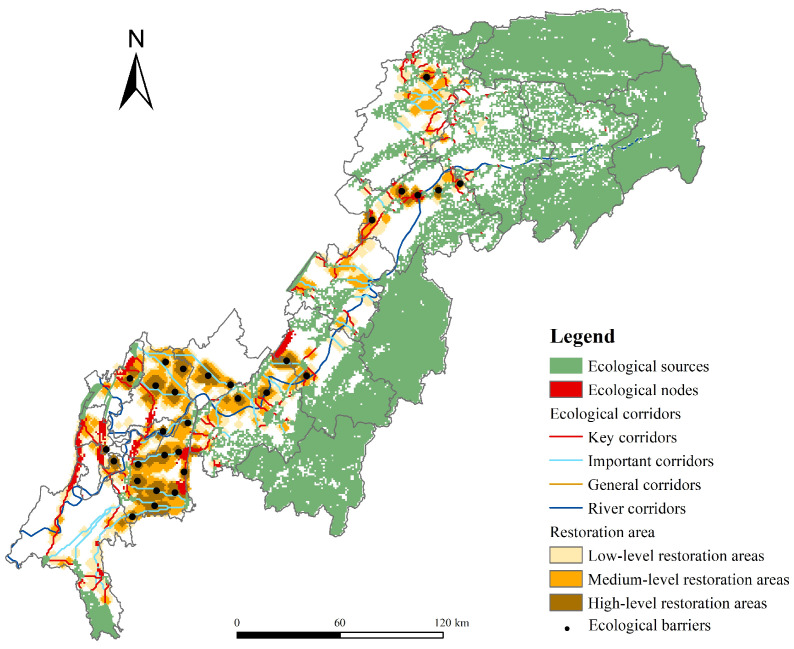
Ecological security patterns of the TGRAC.

ESPs link key ecological elements into a system and manage the landscape with an overall insight, which is conducive to making more systematic and comprehensive conservation policies [16]. ESPs are in line with the sustainable development strategy arrangement of ecosystem protection and environmental governance and have practical significance for guiding ecological protection and civilization construction. The TGRAC not only has unique topography features and the construction background of the Three Gorges project but also is the main part of Chongqing, playing an important role in the socioeconomic development and ecological security of southwest China and the Yangtze River basin [52]. Therefore, in response to improvements in the ecological security of the basin and realizing the coordination between environmental protection and socioeconomic development, the VORSD and circuit theory models were used to construct ESPs (Figure 11). According to the research results of the study, combined with the actual ecological environment of the TGRAC, the relevant land and ecological policy recommendations were put forward.

Ecological patches with high ecosystem health levels and nature reserves were used to identify ecological sources. In general, ecological sources were more prevalent in the east and less prevalent in the west, and the western area was dotted with a lack of spatial continuity, which was not conducive to the improvement of the overall regional ecological status. Nature reserves in the southwest were not fully included in the scope of ecological sources (Figure 6a), such as Simian Mountain, Jinyun Mountain, and Huaying Mountain. Rapid urban expansion not only led to the gradual degradation of the peripheral ecosystem but also reduced the function of providing ESs for urban areas [55]. In the peripheral areas of cities, it is necessary to strictly guard the boundaries of urban development, strengthen the construction of green infrastructure, prohibit development and construction at the expense of ecological damage, and increase the concentration of ecological patches. The results showed that 15.63% of ecological sources belonged to cultivated land. Cultivated land not only plays a significant role in material production, biodiversity maintenance, and climate regulation but also is significantly affected by human activities [56]. The results of the ecosystem health evaluation showed that most of the cultivated land was in general grade, indicating that the ecological function and potential sources of cultivated land should be emphasized.

Most of the ecological corridors with high resistance were concentrated in the ecological sources far apart in the central urban region. The special mountainous terrain and the crossing of construction land led to the high resistance of corridors, especially in the north of Jiangjin, the north of Yubei, and Banan District. In highly urbanized areas, the dramatic impact of human activities will lead to landscape fragmentation, thus affecting the ecological process. Stepping stones as an effective strategy have aroused extensive research [57]. Therefore, it is necessary to increase greenbelts along the roads and improve the green area with reasonable space in highly urbanized areas to strengthen vegetation coverage and connection efficiency. Note that rivers are habitats for species and provide a pathway for species to migrate [52]. Considering the special environment of the TGRAC and the important ecological value of rivers, the Yangtze River and Jialing River should be defined as ecological corridors to improve the corridor network [20]. Ecological nodes were mainly distributed in the construction land and cultivated land in the southwest, which should be included in the restricted construction areas to reduce the interference of human activities. Restoration of ecological barriers was beneficial to improve the connectivity of ecological networks and the ecological environment of central urban areas, especially in Zhongliang Mountain, Causeway Mountain, Shengdeng Mountain, and Mingyue Mountain.

### 5.3. Limitations

We only selected four typical ecosystem services of water production, soil conservation, carbon sequestration, and grain crop production owing to the limitations of data acquisition. This restriction would affect the identification of ecological sources. In future research, we will strengthen the comprehensive measurement of ESs supply and demand to improve the accuracy of ecological sources identification [24]. It is necessary to expand and verify the data sources, strengthen remote sensing and field verification, and optimize the experiments to make model parameters more scientifically robust. Furthermore, given the spillover effects of ecological processes, ecological security is affected by ecological and socioeconomic elements within and outside the region. We will fully consider various factors that affect the construction of ESPs in broader ecological conditions. Finally, the current research on ESPs is about a certain period of time in the past or present. However, regional ecological protection planning is long-term, dynamic, and predictable work [58]. Moreover, land use and climate change have serious impacts on ecological security [59]. Therefore, it is still necessary to discuss ESPs under different land use scenarios or climate scenarios in the future. In this way, the research on ESPs will form a “past-present-future” perspective, which is conducive to ecological civilization construction in a long-term sequence.

## 6. Conclusions

The construction of ESPs is not only conducive to alleviating the contradiction between ecological environmental protection and economic development but also one of the important ways to ensure regional ecological security. However, most previous studies on ESPs mainly focused on ESs supply but did not fully consider the ecosystem health and human demand for ESs. Based on the traditional VORS ecosystem health evaluation framework, this study took ecological demand into account and constructed the VORSD assessment framework to evaluate ecosystem health status to identify ecological sources. The circuit theory was established to identify ecological corridors, nodes, and barriers. Therefore, ESPs of the TGRAC were finally constructed. The following conclusions were obtained: the improved VORSD model could not only reflect the reality of the regional ecosystem more accurately but also account for the benefits to humans. The general ecosystem health of the TGRAC was not optimistic, and the spatial distribution pattern was high in the east and low in the west. A total of 95 ecological sources were identified, with an area of 25,350.16 km^2^, which is mainly distributed in the northeastern and southeastern areas of the TGRAC. Ecological sources mainly included forestland, grassland, and cultivated land, in which forestland played a leading role in all land use types. There were 201 ecological corridors in TGRAC with a total length of 2291.41 km, among which there were 153 key corridors accounting for 76.12% of the total corridors number. In total, 82 ecological nodes were identified, with an area of 629 km^2^. In addition, 30 ecological barriers were extracted and should be considered propriety areas for ecological conservation. The research results verify that ecological sources identified by the VORSD model are more reasonable and provide a complete framework for understanding the complex interactions between natural ecosystems and socioeconomic systems. Meanwhile, the research results are expected to provide guidance for regional ecological protection planning and management measures.

## Figures and Tables

**Table 1:** Methods and processes of the ecosystem physical health assessment.

Correlation Factor	Computational Methods	Description
*EO*	$\begin{array}{l} EO=0.35LH+0.35LC+0.30IC\\ =\left(0.25SHDI+0.10AWMPFD\right)+\left(0.25FN1+0.10CONT\right)\\ +\left(0.07FN2+0.03CONHESION1+0.07FN2+0.03CONHESION2+0.07FN4+0.03CONHESION3\right) \end{array}$	*LH* is landscape heterogeneity; *LC* represents landscape connectivity; *IC* is the connectivity of important ecological function patches.
*ER*	$ER=0.4\times C_{resistan}+0.6\times C_{resilien}$	*C_resilien_* and *C_resistan_* represent the resilience coefficient and resistance coefficient of different land use types.

**Table 2:** Comparison of the results of ecosystem health calculations between the two methods.

	Maximum Value	Average Value	Median Value	Standard Deviation
VORS	0.89	0.61	0.60	0.19
VORSD	0.82	0.56	0.57	0.17

## Data Availability

The data presented in this study are available in the corresponding references.
